# Variability of perfusion dark rim artifacts due to Gibbs ringing

**DOI:** 10.1186/1532-429X-11-S1-P257

**Published:** 2009-01-28

**Authors:** Pedro Ferreira, Peter Gatehouse, Peter Kellman, Chiara Bucciarelli-Ducci, David Firmin

**Affiliations:** 1 Imperial College London, London, UK; 2 Royal Brompton Hospital, London, UK; 3 National Institutes of Health, Bethesda, MD USA

**Keywords:** Myocardial Perfusion Imaging, Perfusion Study, Image Interpolation, Bicubic Interpolation, Zero Filling

## Background and aims

Gibbs ringing is a well known source of Dark Rim Artifacts (DRA) in myocardial perfusion imaging [1]. We examine the variability of this artifact. Specifically, we show that Gibbs artifacts are highly dependent on the edge position and that sub-pixel shifts can dramatically change the appearance.

## Methods

Sub-pixel shifts were introduced in four *in-vivo* raw-data perfusion studies, where a DRA was visible. The shifts had a step of 1/8^th^ of a pixel ranging from 0.125 to 0.875 of the in-plane pixel size. The unprocessed raw-data was phase-shifted using MATLAB before reconstructing it on the scanner using the same image processing as the original data.

The original perfusion study was done on a 1.5 T scanner (Avanto; Siemens): hybrid-EPI sequence with an EPI factor of 4; TR/TE of 5.1/1.7 ms; base resolution 128 pixels; pixel size 2.8 × 2.8 × 8 mm; flip angle 30°; bandwidth 1860 Hz pixel^-1^; TI (time of inversion) of 90 ms using a non-selective BIR-4 saturation pulse, TSENSE with R = 2. Perfusion was imaged during first pass of Gd-DTPA; stress induced by adenosine.

The original and shifted magnitude images were compared visually using CMRtools which applies sub-pixel interpolation in the image space.

The DRAs shown were carefully selected so as to not coincide with any known real perfusion defect.

Sub-pixel shifts in short-axis images were also simulated numerically in MATLAB. Images were also reconstructed with image based bicubic interpolation and zero filling with a factor of 4, and compared.

## Results and discussion

**Figure 1:**
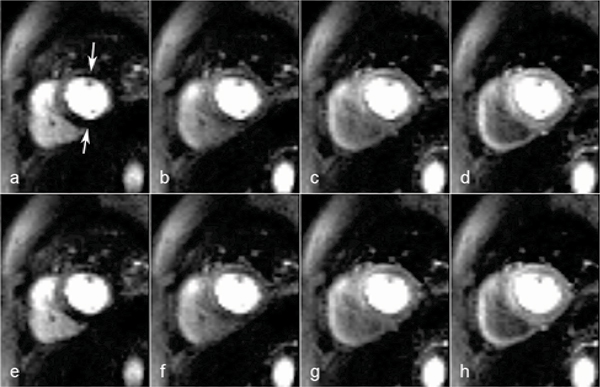
Figure 1

Figure 1 shows 4 consecutive frames during the arrival of the CA into the myocardium of a particular patient, in the basal slice for two different sub-pixel shifts. The top row (*a*-d) shows DRAs in the anterior and inferior segments of the subendocardium (white arrows) for the shift where the artifacts were most prominent (0.125 shift). The bottom row (e-*h*) corresponds to the same frames, but with a half-pixel shift in the vertical direction in relation to the top row (0.625 shift). The DRAs in those regions are highly reduced when compared to the top row.

**Figure 2:**
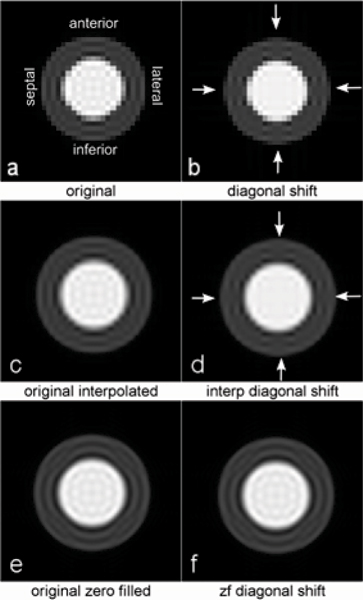
Figure 2

Figure 2a shows the numerically simulated short-axis image with Gibbs artifacts mainly in the septal, inferior, lateral and anterior regions of the subendocardium. After a diagonal shift of half-pixel, Figure 2b shows a reduction of the artifacts in those regions. Figure 2c–d and Figure 2e–f are identical to Figure 2a–b, but with a bicubic image based interpolation and with zero-filling before FFT respectively. Post-FFT image interpolation shows the same dependency on the position of the edge as the non-interpolated data. In contrast, the artifacts appear consistently without regard to the inplane offsets in the zero-filled data, in agreement with Du et al. [2].

Although the shift selected in Figure 1a–d was the worst-case, it should be recognised that this occurs by chance depending on image plane, in-plane offsets, and the patient's respiratory and cardiac motion. This is consistent with the random nature of DRA occurrence in clinical practice.

## Conclusion

The visibility of Gibbs DRAs in perfusion studies is very dependent on the position of the subendocardial wall inside the pixel in the absence of zero-filled pre-FFT interpolation. Position variations from frame to frame in a typical gated perfusion study can explain some of the variability often seen in DRAs. Interpolation by zero-filling prior to enlarged FFT regularizes the DRA appearance.
